# Exploratory Pre–Post Study of School-Based Stress Interventions in Primary School Children

**DOI:** 10.3390/bs15101374

**Published:** 2025-10-09

**Authors:** Isabelle May

**Affiliations:** Department of Educational Psychology and Research on Excellence, Friedrich-Alexander-Universität Erlangen-Nürnberg, Regensburger Straße 160, 90478 Nürnberg, Germany; isabelle.may@fau.de

**Keywords:** school-based intervention, stress, primary school, Yoga, Climbing, social–emotional learning, exploratory study

## Abstract

Background: This exploratory, non-randomized pre–post study compares three school-based stress interventions—Yoga, Climbing, and Social–Emotional Learning—in primary school children. Methods: We compared three low-threshold interventions delivered during regular lessons: (1) a six-week video-guided Yoga sequence (*n* = 64; grade 3), (2) a 2.5-week social–emotional learning (SEL) module focused on emotion recognition and regulation (*n* = 60; grade 3), and (3) a two-week Climbing program implemented with a small special-education sample (*n* = 12). Parallel class-matched controls were included for Yoga and SEL (*n* = 64 and *n* = 60, respectively). A quasi-experimental pre–post design was used. Primary outcomes were overall stress and the emotion subdomains of anger, anxiety, and sadness (SSKJ 3–8); the secondary outcome for the Climbing pilot was general self-efficacy (SWE). Non-parametric statistics (Wilcoxon signed-rank, Mann–Whitney U) and rank-biserial effect sizes (r) were reported with Holm-adjusted α = 0.05. Results: Yoga and SEL produced significant within-group reductions in overall stress and all emotional subdomains (all *p* < 0.001; r = 0.59–0.75) and outperformed their respective controls at post-test (*p* ≤ 0.038; r = 0.22–0.48). Change-score comparisons between Yoga and SEL were not statistically different (*p* ≥ 0.44). In the exploratory Climbing group, self-efficacy increased significantly (V = 64.5, *p* = 0.006, r = 0.80); stress outcomes mirrored Yoga/SEL trends but were under-powered. Conclusions: A brief classroom Yoga routine and a condensed SEL module each yielded clinically meaningful reductions in stress among primary-school pupils, offering flexible options for post-pandemic recovery. Preliminary evidence suggests that Climbing may enhance self-efficacy in older students with psychological challenges; however, larger samples are required. Integrating cost-effective physical and emotional strategies can help schools promote resilience and well-being amid ongoing educational disruptions.

## 1. Introduction

### 1.1. Stress in Childhood and Adolescence

Children and adolescents are increasingly exposed to elevated stress levels, which contribute to rising rates of mental health concerns, somatic complaints, and academic difficulties. According to Lazarus’s (1991) transactional model of stress, stress occurs when perceived situational demands exceed available coping resources. In this framework, primary appraisal (threat/challenge) and secondary appraisal (perceived coping capacity) jointly determine whether situational demands are experienced as stressful. Parental support and monitoring function as protective resources that can shift appraisals and buffer stress exposures at school. In school contexts, such appraisals are frequent and have been linked to diminished well-being, impaired academic performance, and behavioral challenges (Compas et al., 2017; Shonkoff et al., 2012). Schools therefore represent a vital access point for early prevention, particularly for the estimated 70–80% of youth who do not receive adequate mental health support (Thornicroft, 2007).

### 1.2. School-Based Stress Prevention Before COVID-19

Before the COVID-19 pandemic, a wide range of school-based prevention programs targeting stress and emotional well-being had been developed and evaluated. Meta-analyses consistently show small-to-moderate benefits for emotional and behavioral outcomes, with social–emotional learning (SEL) among the most robustly supported approaches (e.g., Durlak et al., 2011), and resilience programs (Juhász et al., 2024; van Loon et al., 2020). Effects tended to be stronger when programs targeted higher-risk students and followed clearly manualized procedures (van Loon et al., 2020; Amanvermez et al., 2023).

### 1.3. The Impact of the COVID-19 Pandemic on Student Stress

For many children—especially those in disadvantaged or otherwise vulnerable circumstances—school routines played a central role in supporting emotion regulation and mental health. Multiple large-scale surveys (2021–2023) documented marked increases in school-related stress and mental-health symptoms during and after the pandemic (Thorn & Vincent-Lancrin, 2021; Helm et al., 2022). For example, the German School Barometer 2024 reports that about 20% of students still experience high psychological strain, rising to roughly 30% among socially or economically disadvantaged groups (Robert Bosch Stiftung, 2024). International reviews estimate that 18–60% of children and adolescents exposed to pandemic-related restrictions exhibited clinically relevant emotional distress (Samji et al., 2022; Madigan et al., 2023). Symptoms included increased anxiety, sadness, frustration, and irritability—often accompanied by sleep problems, concentration difficulties, and psychosomatic complaints (May & Hörl, 2024; Hamaideh et al., 2022; Jiao et al., 2020). The burden was particularly pronounced where safe and stable learning environments were lacking or families faced financial or psychosocial strain. Younger students—especially in early and middle childhood—were more affected due to limited self-regulation capacities and greater dependence on supportive interpersonal contexts (Helm et al., 2022). As schools resumed in-person routines, lingering stress and widened disparities created a need for feasible, embedded prevention formats that fit everyday classroom constraints.

### 1.4. The Need for Post-Pandemic Prevention Formats

Despite the return to in-person schooling, many low-threshold prevention routines have not been reintegrated, while academic demands have reverted to—or exceeded—pre-pandemic levels. This combination has created sustained problem pressure in classrooms, particularly for vulnerable learners. Consequently, there is an urgent need for feasible, scalable, and manualized formats that can be embedded in everyday lessons and delivered with limited staff time and training (Thorn & Vincent-Lancrin, 2021). The present study responds to this need by examining three conceptually distinct, low-intensity approaches (Yoga, SEL, Climbing) under real-world school constraints.

### 1.5. Theoretical Frameworks

The present study examines three low-threshold school formats—Yoga, SEL, and Climbing—selected for feasibility under post-COVID constraints and for their complementary mechanisms within Lazarus’s transactional model of stress. In this model, stress arises when perceived demands exceed coping resources via primary (threat/challenge) and secondary (coping capacity) appraisal.

Body- and attention-based routines (Yoga) can down-regulate arousal, enhance interoception, and sharpen attentional control, helping pupils appraise everyday demands as less threatening. School studies report small-to-moderate improvements in stress and negative affect, particularly when brief practices are embedded in class time (e.g., Bazzano et al., 2018; Pascoe et al., 2017; Ferreira-Vorkapic et al., 2015; Telles et al., 2019). SEL curricula strengthen emotion labeling, reappraisal, and problem-solving, thereby expanding coping resources; large meta-analyses show robust benefits for emotional and behavioral outcomes (e.g., Durlak et al., 2011). Climbing emphasizes graded mastery, goal progression, peer support, and immediate feedback—conditions that foster self-efficacy in Bandura’s (1997) framework and can buffer stress in youth (e.g., Gerber et al., 2017; Mazzoni et al., 2009).

Synthesizing these accounts, Yoga chiefly targets primary appraisal by reducing perceived demands through physiological calm and attentional control; SEL targets secondary appraisal by enlarging the cognitive–verbal coping repertoire; and Climbing targets secondary appraisal by building self-efficacy via mastery and modeling. We therefore expect convergent stress-related benefits through distinct mechanisms. Given the non-randomized pre–post design and heterogeneous dose, these expectations are framed as exploratory and hypothesis-generating, not confirmatory.

Finally, in Bandura’s social–cognitive theory, self-efficacy—beliefs about one’s capability to organize and execute actions—shapes initiation, persistence, and recovery in effortful tasks. In the appraisal model, stronger efficacy beliefs contribute to secondary appraisal by increasing perceived coping capacity and, in turn, lowering stress responses. In our context, Climbing most directly cultivates efficacy via mastery and feedback; SEL supports efficacy for cognitive coping through guided practice in labeling/reappraisal; and Yoga supports efficacy indirectly via regulation of physiological and affective states.

### 1.6. Rationale and Research Questions

There is a clear need for feasible, low-threshold school interventions that are theoretically grounded and workable under post-COVID constraints. We explicitly frame the present work as exploratory due to its non-randomized pre–post design and small/unequal subgroups. The study focuses on three conceptually distinct formats—Yoga, SEL, and therapeutic Climbing—because they target complementary mechanisms of stress regulation (somatic/interoceptive calm; cognitive–verbal coping; mastery/agency) and can be embedded in everyday lessons with limited resources. Comparative evidence within primary education remains scarce, which motivates the present quasi-experimental evaluation.

The primary outcome was overall stress (SSKJ 3–8). Secondary outcomes comprised the discrete emotion subdomains—anger, anxiety, and sadness—for the classroom arms, and general self-efficacy for the exploratory Climbing arm.

Accordingly, we posed the following research question: “Do Yoga, SEL, or Climbing reduce stress among primary-school children after COVID-19, and which mechanisms are most plausibly involved?”

The primary aims of the study were:

(1) To evaluate pre–post changes in overall stress (primary) and discrete emotions (secondary) for Yoga and SEL in primary classrooms.
(2) To conduct a preliminary exploration of Climbing in a special-education context with self-efficacy as a key (secondary) outcome.
(3) To derive practice-oriented implications for scalable, post-pandemic school mental-health support.

From this rationale, we derived the following explanatory hypotheses:

**H1 (Yoga).**  *Participants will show pre–post reductions in overall stress, consistent with evidence that school-based mindfulness/Yoga improves stress and negative affect via interoceptive regulation and calm attention (e.g., Pascoe et al., 2017; Telles et al., 2019).*

**H2 (SEL).**  *Participants will show pre–post reductions in overall stress and in anger, anxiety, and sadness, in line with meta-analytic findings that SEL strengthens cognitive and verbal emotion-regulation skills (Durlak et al., 2011).*

**H3 (Yoga vs. SEL).**  *Given their distinct mechanisms (somatic/interoceptive vs. cognitive–verbal), we expect potentially different change patterns across outcomes, without assuming equivalence or superiority.*

**H4 (Climbing, exploratory).**  *Participants will show a pre–post increase in general self-efficacy, consistent with Bandura’s model of mastery and social modeling and prior work on mastery-oriented physical activities (Bandura, 1997; Gerber et al., 2017; Mazzoni et al., 2009).*

These hypotheses are anchored in Lazarus’s transactional model (primary vs. secondary appraisal; Lazarus, 1991) and Bandura’s (1997) self-efficacy theory, providing a theory-consistent, practice-proximal rationale for testing body-based, cognitive–verbal, and mastery-oriented routes to stress reduction in primary schools.

## 2. Materials and Methods

### 2.1. Sample

A total of 260 primary school children (Grades 3–4; ages 8–9 years) participated across three formats: Yoga (*n* = 64) with a class-matched control (*n* = 64), SEL (*n* = 60) with a class-matched control (*n* = 60), and an exploratory single-group Climbing arm (*n* = 12) in a special-education context.

For the matched comparisons (Yoga; SEL), intact same-grade parallel classes from the same school were allocated to intervention or control prior to baseline. To reduce selection bias and enhance comparability, we ensured similar school contexts (school type/track, region) and cohort characteristics (grade level; available socioeconomic indicators from the Bavarian Education Report, 2024 prior exposure to mental-health programs) (Bavarian State Ministry of Education and Cultural Affairs, 2024). No individual randomization or formal propensity-score matching was performed. This class-level allocation reflects typical school practice and increases ecological validity; given the lack of individual randomization, all inferences are interpreted as exploratory.

The three interventions differed in duration due to timetable constraints and inherent format characteristics (Yoga as a brief daily micro-practice; SEL as a condensed workshop; Climbing as a therapeutic block). We therefore treat duration as a contextual (non-experimental) variable and report it descriptively; implications are addressed in the limitations.

**Figure 1:**
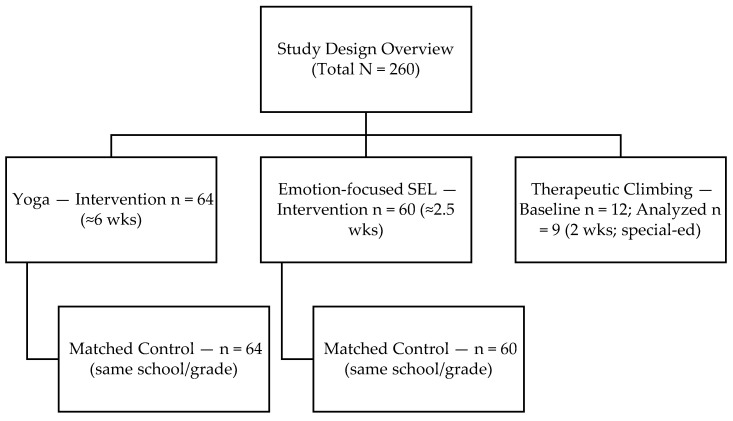
Study design overview.

**Figure 2:**
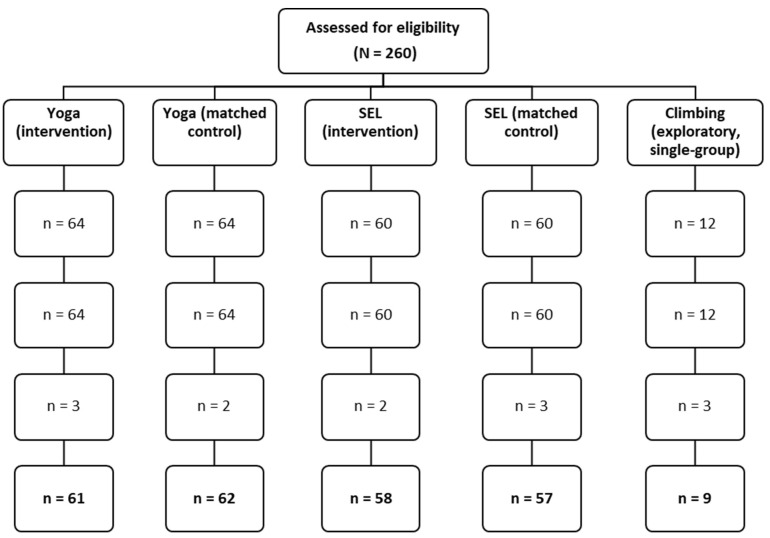
CONSORT-style flow of recruitment, class-level allocation, post-test attrition, and analyzed samples. Rows indicate Allocated, Received intervention/Usual classes, Lost to follow-up (post), and Analyzed (complete pre–post). Allocation occurred at the class level (no individual randomization); matched controls received usual classes. The Climbing arm is single-group and exploratory.

Climbing (exploratory arm). The Climbing group comprised *n* = 12 students (33% [4/12] girls) from a special-education setting; documented diagnoses included generalized anxiety disorder and ADHD. The intervention differed in setting and content, being delivered over two weeks in a Climbing facility with individual belaying and structured transfer activities. Given the small size and contextual specificity, analyses are within-group pre–post only; no direct comparisons are made to the Yoga or SEL arms. Of the 12 enrolled, *n* = 9 provided complete pre–post data for analysis (study overview in Figure 1, attrition/missingness detailed in Figure 2).

Baseline equivalence for the matched comparisons was examined using Mann–Whitney U for continuous/ordinal variables (age; baseline SSKJ overall stress) and χ^2^ (Fisher’s exact where expected counts < 5) for proportions (girls). No significant baseline differences were observed (Table 1a). Baseline descriptives for the Climbing arm are presented separately (Table 1b, descriptive only).

Figure 1 provides a study design overview. Two class-matched comparisons (Yoga vs. control; SEL vs. control) and one exploratory single-group Climbing arm. Allocation occurred at the class level (intact, same-grade parallel classes within the same school); no individual randomization and no formal propensity-score matching were performed. Comparability was ensured via school context and cohort similarity (including indicators from the Bavarian Education Report, 2024). Primary outcome: Overall stress. Secondary outcomes: Discrete emotions (anger, anxiety, sadness) and, for Climbing, general self-efficacy. Given the non-randomized design and small/unequal subgroups, all inferences are exploratory.

### 2.2. Measures

The primary outcome was overall stress. Secondary outcomes included the discrete emotion subdomains—anger, anxiety, and sadness—and, for the Climbing arm, general self-efficacy.

Stress and discrete emotions were assessed with the SSKJ 3–8 (Stress and Coping Questionnaire for Children; Lohaus et al., 2018). Acute school-related stress and associated negative emotions were assessed with the SSKJ 3–8. To accommodate classroom time and maintain feasibility, we employed a short school-focused form comprising 18 of the original 30 items, covering overall stress as well as anger, anxiety, and sadness. Items refer to recent situations at school and are answered on a 5-point Likert scale from 1 = “never” to 5 = “very often”; higher scores indicate higher levels of the respective construct. Administration took place in class groups by trained staff with standardized instructions; reading support was provided where necessary. For each subscale and the overall stress index we computed mean scores from available items; participants with ≥80% completed items contributed to the score, and up to 20% missing items were imputed by the person-specific mean of completed items on that subscale; otherwise, the subscale was set to missing. Internal consistencies in the present sample were α = 0.86 (overall stress), α = 0.79 (anger), α = 0.80 (anxiety), and α = 0.74 (sadness). Prior work supports factorial stability and age-appropriateness of the SSKJ 3–8 for children aged 8–10 years (Lohaus et al., 2018; Eschenbeck et al., 2020).

#### 2.2.1. General Self-Efficacy (SWE; Schwarzer & Jerusalem, 2003)

In the Climbing arm, general self-efficacy—defined as the belief in one’s capability to organize and execute actions required to manage demands—was measured with the 10-item SWE scale using a 4-point response format (1 = “not at all true” to 4 = “exactly true”); higher values reflect stronger self-efficacy. The scale was administered in classrooms with the same standardized procedures as above. Scores were computed as the mean of items applying the same missing-data rule (≥80% completion; person-mean substitution up to 20% missing). Internal consistencies were α_pre = 0.87 and α_post = 0.89. The inclusion of self-efficacy is theoretically motivated by its role in secondary appraisal (Bandura, 1997), which is targeted by mastery- and feedback-oriented Climbing activities.

#### 2.2.2. Demographics and Context Variables

We recorded age, gender (girl/boy/other), migration background (yes/no), family structure (single-parent, two-parent, other), and prior participation in extracurricular prevention programs (yes/no). Where available, school-level socioeconomic indicators from the Bavarian Education Report (2024) informed class pairing (see Section 2.1).

Finally, all measures were collected pre and post within regular class time. Reporting follows the analysis plan in Section 2.5 (non-parametric tests with justified α-control and effect sizes; exact *p*-values).

### 2.3. Intervention Protocols

#### 2.3.1. Overview and Implementation Context

All three formats were delivered during regular school hours using structured manuals and standardized procedures. Interventionists were qualified practitioners: the Yoga sessions were led by a certified children’s Yoga teacher; the SEL lessons were taught by a trained educational professional with SEL program training; the Climbing block was conducted by a licensed Climbing therapist/certified Climbing trainer in collaboration with a school psychologist. To support procedural fidelity, each format used a brief session checklist and an attendance log; deviations from the manual were noted, and leaders had access to scripted prompts (for relaxation/imagery in Yoga; for role-play and reflection questions in SEL; for transfer questions in Climbing). Table 2 provides an overview of the interventions.

#### 2.3.2. Yoga (6 Weeks, Classroom-Embedded Micro-Practice)

The Yoga protocol consisted of three 15-min sessions per week over six weeks (brief breathing, age-appropriate Hatha postures, and short guided relaxation). Weeks 1–2 emphasized modelling and full verbalisation of movements; Weeks 3–6 consolidated breath awareness and a short flow repeated across sessions, with a singing bowl to cue transitions. A one-minute reflection at the end (“How do I feel now?”) supported metacognitive awareness. Delivery followed a step-by-step manual including audio scripts for the relaxation phases; students indicated their state on a simple feeling board after each session.

#### 2.3.3. SEL (2.5 Weeks, Scripted Workshops)

The SEL module comprised three 45-min lessons per week for 2.5 weeks and targeted (a) recognizing and naming feelings, (b) managing anger and frustration (including a brief “Stop-and-Think” routine and a feelings diary), and (c) coping with anxiety and sadness (balloon breathing, brief progressive muscle relaxation, and small-group discussion of helpful strategies). Content and phrasing were fully scripted in the SEL program guide to standardise delivery across classes.

#### 2.3.4. Climbing (2 Weeks, Exploratory Single-Group)

The Climbing block consisted of two 90-min units per week for two weeks in a Climbing facility (~60 min Climbing + 30 min structured debrief per unit). Students worked in pairs (one belaying, one Climbing) under therapist supervision to cultivate trust and graded mastery; debriefs used fixed prompts (e.g., “When did you feel most proud?” “What helped you when you felt fear?”) to support self-efficacy, peer support, and emotion regulation. All participants had medical clearance and were familiar with safety procedures. Given the small, special-education sample and single-group design, analyses are exploratory (see Section 2.1 and Section 2.5).

#### 2.3.5. Dose and Fidelity Considerations

Because school timetables and format characteristics differed, intended dose varied across arms (Yoga ≈ 18 sessions × 15 min ≈ 270 min; SEL ≈ 7–8 sessions × 45 min ≈ 315–360 min; Climbing ≈ 4 sessions × 90 min ≈ 360 min). We therefore treat duration as a contextual variable rather than an experimental factor and monitored attendance-based exposure and fidelity throughout. See Supplement S1 for the monitoring instruments and procedures; aggregate summaries are available upon request. This framing prevents over-interpretation of between-format contrasts while preserving ecological validity.

### 2.4. Data Analysis

All analyses were conducted in R 4.4.0 (R Core Team, 2024) using dplyr and rstatix. Because outcomes are ordinal/approximately ordinal (5-point scales) and subgroup sizes are small/unequal, we prespecified rank-based non-parametric tests. We used a complete-case approach (participants with both pre- and post-data; analyzed n per arm in Figure 2). For each test family we report exact two-sided *p*-values, effect sizes with 95% CIs (bootstrap, 2000 resamples), and medians [IQR].

Multiple testing control: To avoid over-conservatism, we applied Holm adjustments (α = 0.05, two-sided) within families: (a) Yoga within-group outcomes; (b) SEL within-group outcomes; (c) Yoga vs. SEL between-arm comparisons (overall stress and emotion subscales). Exploratory Climbing analyses were not α-adjusted beyond the single test per outcome and are interpreted cautiously.

Effect sizes: For within-group Wilcoxon signed-rank tests we report r = Z/√Npairs; for between-group Mann–Whitney U tests we report rank-biserial r (and provide Cliff’s δ in the Supplement S2 as a robustness descriptor). Where helpful for practice interpretation, we add paired Cohen’s dz for within-group change (Supplement S2).

Sensitivity/robustness checks: Given unequal subgroup sizes and potential ties, we pre-specified (i) Hodges–Lehmann median change estimates with 95% CIs and (ii) a robust rank-based ANOVA on change scores (aligned ranks) as a sensitivity analysis for H3 (reported in Supplement S2). Results did not alter conclusions.

#### 2.4.1. Within-Group Changes (H1, H2)

For Yoga (H1), we tested pre–post change in overall stress using Wilcoxon signed-rank tests on paired data (analyzed n per Figure 2). For SEL (H2), we tested pre–post changes in overall stress and discrete emotions (anger, anxiety, sadness) using Wilcoxon signed-rank tests, with Holm adjustment across the four outcomes. We report medians [IQR], r (95% CI), and exact *p*.

#### 2.4.2. Between-Arm Comparison: Yoga vs. SEL (H3)

To examine whether change patterns differed between Yoga and SEL, we computed Δ scores (post–pre) for overall stress and each emotion subscale and compared arms using Mann–Whitney U tests (Holm-adjusted within this family). As a sensitivity analysis we ran a rank-based ANOVA on aligned ranks for Δ scores (reported in Supplement S2). The Climbing arm was not included in H3.

#### 2.4.3. Exploratory Single-Group Analysis: Climbing (H4)

Within the Climbing arm, we assessed pre–post change in general self-efficacy (SWE) using the Wilcoxon signed-rank test (analyzed *n* = 9). Given the small, single-group design, these findings are exploratory; we present medians [IQR], r (95% CI), and exact p, and refrain from cross-arm claims.

#### 2.4.4. Summary Table of Hypotheses, Variables, and Tests

Table 3 maps hypotheses to outcomes, procedures, comparisons, and effect sizes; example R code and exact formulas are provided in the Supplement S2.

### 2.5. Statistical Reporting, Missing-Data Handling, and Robustness

Analyses followed a pre-specified reporting plan to ensure transparency and comparability across arms. We used complete-case datasets for each test (participants with both pre- and post-measurements; analyzed n per arm shown in Figure 2). For every outcome we report medians [IQR], exact two-sided *p*-values, and effect sizes with 95% confidence intervals.

Missing data: Subscale scores were computed if ≥80% of items were completed; up to 20% missing items within a subscale were imputed by the person-mean of completed items; otherwise, the subscale was set to missing for that wave. Individuals with missing pre or post on a given outcome were excluded from that outcome’s paired test (complete-case). We summarize missingness and reasons (e.g., illness/absence) in Figure 2. Supplement S1 documents the monitoring instruments and procedures used for attendance, fidelity, and deviation logging.

Multiple testing and α-control: The familywise error rate was controlled using Holm adjustments (α = 0.05, two-sided) within test families: (a) Yoga within-group outcomes; (b) SEL within-group outcomes; (c) Yoga-vs-SEL between-arm Δ-comparisons. The Climbing arm comprised exploratory single-group tests and was not additionally multiplicity-corrected beyond the single outcome per test; interpretation is cautious and hypothesis-generating.

Effect sizes and CIs: For Wilcoxon signed-rank tests we report r = Z/√Npairs; for Mann–Whitney U we report rank-biserial r (and provide Cliff’s δ in Supplement S2). To complement rank tests, we report Hodges–Lehmann median change (or median difference in Δ) with 95% CIs. Where informative for practice, paired Cohen’s dz is added in Supplement S2. Confidence intervals for effect sizes were obtained via bootstrap (2000 resamples) with bias-corrected and accelerated limits.

Assumptions and sensitivity analyses: Outcomes are ordinal (5-point) and group sizes small/unequal; we therefore use rank-based methods throughout. For H3 we conducted a robust rank-based ANOVA on aligned ranks for Δ-scores as a sensitivity check alongside the primary Mann–Whitney comparisons; conclusions were unchanged (see Supplement S2). Ties and zero differences were handled with exact or large-sample approximations as appropriate (reported by test).

Software, reproducibility, and transparency: Analyses were run in R 4.4.0 with dplyr and rstatix (Section 2.4). All scripts are annotated and will be shared on the Open Science Framework (OSF) with the anonymized dataset and a session-info file to ensure reproducibility; the OSF DOI will be provided during peer review.

Interpretation policy: Given class-level allocation (no individual randomization) and unequal doses across formats, between-arm findings are interpreted exploratorily and with emphasis on effect sizes and precision rather than dichotomous significance. We refrain from cross-format efficacy claims for the Climbing arm (single-group).

## 3. Results

### 3.1. Yoga Intervention (H1)

We examined pre–post change in the Yoga arm using Wilcoxon signed-rank tests on complete pairs (analyzed n as in Figure 2). Across all SSKJ outcomes, median levels decreased significantly from pre to post (Table 4). Effect sizes (r) were large (r ≥ 0.59).

All effects were significant (r ≥ 0.59).

Between-Group Comparisons (Yoga vs. Yoga Control at Post-Test)

At post-test, stress-related symptoms were significantly lower in the Yoga group compared to the control group, as indicated by Mann–Whitney U tests (Table 5).

### 3.2. SEL (H2)

Within the SEL arm, Wilcoxon signed-rank tests showed significant pre–post reductions for overall stress and all emotion subscales (Table 6). Effect sizes were large (r ≈ 0.62–0.64).

Between-group (post-test). At post-test, the SEL class showed lower symptom levels than its matched control (Mann–Whitney U; Table 7).

Negative r indicates that the SEL group had lower ranks (i.e., fewer symptoms) than its control group.

### 3.3. Yoga vs. SEL (H3)

To test whether change patterns differed by format, we compared Δ scores (post–pre) between Yoga and SEL using Mann–Whitney U tests with Holm adjustment within this family (overall stress; anger; sadness; anxiety). No adjusted contrasts reached significance (Table 8).

### 3.4. Climbing (H4, Exploratory)

Within the Climbing arm (single-group), self-efficacy (SWE) increased significantly from pre to post (Wilcoxon signed-rank: V = 64.5, *p* = 0.006, r = 0.80). Given the small sample and absence of a control, these results are exploratory and should be interpreted as hypothesis-generating rather than confirmatory. Descriptive statistics (medians, IQRs) for all outcomes are provided in the Supplementary S2.

## 4. Discussion

### 4.1. Summary of Key Findings

Across both classroom programs, the Yoga and SEL formats showed significant within-group reductions in overall stress and in the discrete emotion domains (anger, sadness, anxiety). Post-test comparisons against matched controls converged on the same pattern, indicating lower symptom levels in the intervention classes. In the direct comparison of change scores (H3), no between-format differences reached significance after multiplicity control. We therefore refrain from claims of superiority or equivalence between Yoga and SEL. Instead, we interpret the results as broadly comparable improvements of similar magnitude under real-world classroom conditions, as reflected by the effect sizes and their confidence intervals. This pattern aligns with meta-analytic evidence that structured, active, school-embedded prevention formats can yield small-to-moderate benefits for stress-related outcomes (e.g., van Loon et al., 2020; Amanvermez et al., 2023).

The exploratory Climbing arm, delivered to a small special-education sample, showed a significant pre–post increase in general self-efficacy. Given the single-group design and limited n, these findings are preliminary and hypothesis-generating. They are nonetheless consistent with the idea that mastery-oriented, physically challenging activities can strengthen coping resources in vulnerable student populations.

Interpretive note: Because dose and implementation constraints differed across formats (Section 2.3), and allocation occurred at the class level, we emphasise effect sizes and precision over dichotomous significance testing when comparing formats.

### 4.2. Interpretation and Theoretical Implications

The results are consistent with Lazarus’ transactional model: stress decreases when appraisals shift toward lower perceived demands and/or greater perceived coping resources. The three formats target complementary leverage points in this process, which plausibly accounts for the observed improvements.

For Yoga, brief classroom routines that induce calm, enhance interoceptive awareness, and narrow attentional focus likely act on primary appraisal, helping pupils to reframe routine stressors as less threatening. This mechanism aligns with school-based evidence for mindfulness-/Yoga-infused micro-practices producing small-to-moderate reductions in stress and negative affect when embedded as short, repeated elements in lessons (e.g., Pascoe et al., 2017; Telles et al., 2019).

For SEL, explicit emotion labeling, reappraisal, and problem-solving broaden secondary-appraisal resources, which coheres with meta-analytic findings of robust SEL benefits for emotional outcomes and school behavior (Durlak et al., 2011). The present reductions in overall stress and discrete emotions are consistent with this pathway.

For therapeutic Climbing, graded mastery experiences, peer modeling, and somatic feedback directly target self-efficacy, a central determinant of secondary appraisal in Bandura’s (1997) framework. Prior work links mastery-oriented, physically demanding activities to perceived competence and stress buffering in youth (e.g., Gerber et al., 2017; Mazzoni et al., 2009), which is compatible with the self-efficacy gains observed here.

Taken together, the convergence of mechanism–task fit and consistent delivery appears more decisive than sheer exposure time under post-pandemic school constraints. In other words, when low-threshold routines are tightly aligned with their putative mechanisms (somatic/interoceptive for Yoga; cognitive–verbal for SEL; mastery/agency for Climbing) and delivered with fidelity, meaningful pre–post changes are achievable even at modest dose. Domain-specific nuances (e.g., broader emotional relief under SEL; strong self-efficacy signal under Climbing) are compatible with mechanism profiles and do not imply categorical superiority of one format over another.

### 4.3. Practical Implications for Schools

For schools, the key message is pragmatic rather than prescriptive. Both classroom formats—Yoga and SEL—produced broadly comparable improvements in stress-related outcomes under real-world conditions. Rather than privileging one approach, schools can select the format that best fits local capacity, timetable constraints, and student needs, while maintaining delivery fidelity.

A mechanism–need match is a useful guide. Brief classroom Yoga routines are well suited when teachers seek a low-threshold, time-efficient practice that cultivates calm attention and interoceptive regulation (e.g., homeroom starters, short PE inserts). SEL is appropriate where students particularly benefit from explicit instruction in emotion labeling, reappraisal, and problem-solving, for example, in counselor-led lessons or when classes struggle with verbal regulation. In practice, schools can alternate formats across terms, run them in parallel across subjects, or target them to different grades or risk profiles; the aim is fit and consistency, not formal equivalence.

Implementation can remain light-touch yet systematic. Yoga can be embedded as 10–15-min micro-sessions delivered by staff with brief training and supported by standardized scripts and audio prompts. SEL can be organized as scripted 45-min lessons taught by trained educational professionals or school psychologists. In both cases, fidelity tools (attendance logs, one-page checklists, fixed closing reflections) help ensure consistent delivery, and simple monitoring (e.g., brief stress check-ins or termly pre–post questionnaires) allows teams to track change without overburdening teachers.

The Climbing arm—although exploratory and small—suggests a complementary pathway for students with special educational needs or pronounced anxiety, where mastery-based, physically challenging activities can strengthen self-efficacy. Schools with access to Climbing or similar provision may consider short, structured blocks with a standardized debrief to translate mastery experiences into coping language.

Taken together, the findings support a portfolio approach: adopt short, scalable routines that align with their putative mechanisms, deliver them with fidelity, and monitor outcomes with simple, age-appropriate tools. Under post-pandemic constraints, this strategy enables schools to build supportive classroom rhythms and incrementally improve students’ stress regulation without demanding extensive new resources.

### 4.4. Limitations

This study should be interpreted as exploratory and hypothesis-generating. Allocation occurred at the class level without individual randomization or blinding, which limits internal validity and leaves room for unmeasured class- or teacher-level influences (i.e., clustering). Outcomes were assessed only in the short term; the durability of effects beyond the immediate post-test is unknown. All outcomes relied on student self-reports, which can be affected by social desirability and comprehension; while self-report is standard for subjective emotional states in school-age children, future trials should add multi-informant and/or behavioral indicators (e.g., teacher ratings, brief attention/arousal proxies).

Dose and format varied by arm (≈6 vs. 2.5 vs. 2 weeks), complicating cross-format attribution; we therefore refrained from dose-based claims and focused on effect sizes and precision rather than dichotomous significance. The Climbing arm was small and single-group (*n* = 12; *n* = 9 analyzed) and thus underpowered and not controlled; findings are preliminary and intended to inform subsequent designs. Data-protection constraints prevented planned moderator analyses (e.g., gender, SES, prior adversity), and results may not generalize beyond the regional school context studied.

Despite these limitations, the study provides initial practice-proximal evidence that brief, structured classroom routines can improve stress-related outcomes under post-pandemic constraints. A logical next step is a cluster-randomized trial with standardized dose, multi-informant outcomes, fidelity auditing, and prespecified moderators to test mechanisms and sustainability more rigorously.

### 4.5. Future Directions

Future work should combine greater internal validity with ecological feasibility. In school settings, cluster-randomized designs at the class or grade level with blinded outcome assessment are a pragmatic next step to strengthen causal inference while respecting timetables. Extending observation windows with follow-ups at 3–6 months (and, where feasible, a second follow-up) will clarify maintenance and delayed effects.

Outcome assessment should move beyond single-informant self-report. Multi-informant batteries (student, teacher, parent) and low-burden physiological or behavioral proxies (e.g., brief attention tasks, simple arousal indices) can triangulate change. To test theorized pathways, studies should include mechanism measures aligned with each format (e.g., interoception/attentional control for Yoga; reappraisal/problem-solving for SEL; mastery experiences and self-efficacy for Climbing) and analyze mediators alongside outcomes.

Given heterogeneous exposure in real schools, future trials ought to standardize dose bands (e.g., predefined session counts/durations) and implement fidelity auditing (attendance, adherence checklists, occasional live/recorded ratings). Sensitivity analyses that adjust for attended dose can help separate implementation from content effects. Where formats show similar magnitudes, equivalence or non-inferiority designs may be appropriate to test whether schools can choose by context-fit without sacrificing impact.

The Climbing format warrants larger, controlled studies in special- and mainstream-education samples, ideally with active comparators and structured debriefs to optimize transfer from mastery experiences to coping language. Mixed-methods designs (short interviews, teacher field notes) can capture experiential and relational ingredients that quantitative scales may miss.

Finally, research should address for whom and under which conditions these interventions work best. Moderator analyses (e.g., gender, SES, baseline regulation, prior adversity) and equity-focused sampling can guide tailoring. Implementation-science outcomes—acceptability, feasibility, cost, and training requirements—will inform scale-up. Pre-registration, open materials/data (OSF), and transparent analysis plans should be standard to enhance reproducibility and cumulative learning.

## 5. Conclusions

Brief, low-threshold school interventions can meaningfully improve students’ emotional well-being in post-pandemic classrooms. In our trial, both the Yoga-based and the SEL programs yielded broad, within-group reductions in overall stress, anger, anxiety, and sadness, suggesting that body-based and cognitive–verbal routes can produce broadly comparable improvements in self-regulation under real-world conditions. Accordingly, schools can select or combine formats based on local resources and student needs, provided delivery is consistent and manualized, without extensive training or external personnel.

The exploratory Climbing pilot adds a complementary perspective: mastery-oriented physical experiences may bolster general self-efficacy in vulnerable learners. Given the small, uncontrolled sample, this signal requires confirmation in larger, controlled studies.

More broadly, embedding scalable, evidence-informed routines into everyday lessons positions schools as key public-health actors and advances educational equity by reaching many students with minimal burden. Investing in short, feasible prevention formats is not ancillary to schooling—it helps create the conditions in which learning, inclusion, and resilience can flourish.

## Figures and Tables

**Table 1:** (**a**) Baseline characteristics (Yoga & SEL matched comparisons). (**b**) Baseline descriptives for the Climbing arm (single-group, exploratory).

(a)						
**Variable**	**Yoga *n* = 64**	**Yoga Control** ***n* = 64**	***p***	**SEL** ***n* = 60**	**SEL Control** ***n* = 60**	***p***
Age, yrs (M ± SD)	8.9 ± 0.4	8.8 ± 0.5	0.17	8.8 ± 0.4	8.7 ± 0.5	0.22
Girls % (n/N)	44% (28/64)	42% (27/64)	0.76 †	50% (30/60)	48% (29/60)	0.83
Baseline SSKJ Overall Stress (Median [IQR])	2.5 [2.0–3.0]	2.4 [2.0–3.1]	0.64	2.4 [1.9–2.9]	2.4 [1.9–3.0]	0.91
Values are M ± SD or Median [IQR]. *p*-values from Mann–Whitney U (continuous/ordinal) and χ^2^ (Fisher’s exact when expected counts < 5) for matched comparisons. Climbing is shown descriptively (no control). † χ^2^ test (Fisher’s exact when expected counts < 5).						
**(b)**						
**Variable**			**Climbing (*n* = 12)**			
Age, years			8–10 (range)			
Girls, % (n/N)			33% (4/12)			
General self-efficacy (SWE)—M ± SD			27.9 ± 4.14			
General self-efficacy (SWE)—Median [IQR]			26.5 [25.8–30.8]			
Values are M ± SD or Median [IQR] where available; no between-group tests are reported. Baseline *n* = 12; analyzed pre–post *n* = 9.						

**Notes.** All participants were from a special-education setting; baseline *n* = 12, analyzed pre–post *n* = 9. The Climbing arm is analyzed exploratorily with within-group tests only; see Section 2.3 for protocol details.

**Table 2:** Overview of interventions (format, dose, staffing, manuals).

Intervention	Duration	Frequency	Session Length	Instructor(s)	Manual/Scripting
Yoga	6 weeks	3×/week	15 min	Certified children’s Yoga teacher	Yes—Standardized school Yoga manual (scripted)
SEL	2.5 weeks	3×/week	45 min	Trained educational professional (SEL-trained)	Yes—SEL program guide (scripted)
Therapeutic Climbing	2 weeks	2×/week	60 min Climbing + 30 min debrief	Licensed Climbing therapist/certified Climbing trainer + school psychologist	Yes—Climbing manual (with debrief prompts)

**Notes.** Attendance and fidelity were monitored via session checklists, and protocol deviations were logged. See Supplement S1 for instruments and procedures.

**Table 3:** Overview of hypotheses, outcome variables, and statistical tests.

Hypothesis	Variable(s)	Statistical Test	Comparison	Effect Size
H1	Overall stress (SSKJ 3–8)	Wilcoxon signed-rank	Yoga: pre vs. post	*r* (95% CI)
H2	Overall stress; anger; anxiety; sadness (SSKJ 3–8)	Wilcoxon signed-rank (Holm within family)	SEL: pre vs. post	*r* (95% CI)
H3	Δ overall stress; Δ anger; Δ anxiety; Δ sadness	Mann–Whitney U (Holm within family); sensitivity: aligned-ranks ANOVA	Yoga vs. SEL	rank-biserial *r* (95% CI); δ (Supp.)
H4	Self-efficacy (SWE)	Wilcoxon signed-rank	Climbing: pre vs. post (expl.)	*r* (95% CI)

**Table 4:** Yoga intervention: Pre–post change (Wilcoxon signed-rank). Values are Medians [IQR]. Exact two-sided *p*-values. Effect size r = Z/√Npairs.

Variable (SSKJ)	Pre Median [IQR]	Post Median [IQR]	V	Z	*p*	r
Overall stress	2.5 [2.0–3.0]	1.8 [1.2–2.4]	1788	−6.12	<0.001	0.72
Anger	2.3 [1.8–2.9]	1.6 [1.0–2.2]	1659	−5.09	<0.001	0.60
Sadness	2.0 [1.5–2.4]	1.4 [1.0–1.8]	1815	−6.38	<0.001	0.75
Anxiety	2.1 [1.6–2.6]	1.5 [1.0–2.0]	1656	−5.02	<0.001	0.59

**Table 5:** Yoga vs. Control (post-test, Mann–Whitney U).

Variable	U	Z	*p*	r
Overall stress	1570.5	−2.28	0.023	0.23
Anger	1064.0	−5.04	<0.001	0.48
Sadness	1495.5	−2.61	0.009	0.27
Anxiety	1293.0	−3.96	<0.001	0.37

**Notes.** Negative Z/r indicate lower ranks in the Yoga group (i.e., fewer symptoms) relative to control. Two-sided tests; no multiplicity adjustment within this post-hoc contrast family.

**Table 6:** SEL intervention: Pre–post change (Wilcoxon signed-rank).

Variable	Pre Median [IQR]	Post Median [IQR]	V	Z	*p*	r
Overall stress	2.4 [1.9–2.9]	1.7 [1.2–2.3]	1440.5	−5.35	<0.001	0.64
Anger	2.2 [1.7–2.7]	1.5 [1.0–2.1]	1417.0	−5.13	<0.001	0.62
Sadness	2.1 [1.6–2.6]	1.4 [1.0–1.9]	1427.0	−5.13	<0.001	0.62
Anxiety	2.0 [1.5–2.5]	1.3 [0.9–1.8]	1445.5	−5.40	<0.001	0.64

**Table 7:** SEL vs. control at post-test (Mann–Whitney U).

Variable	U	Z	*p*	r
Overall stress	1724.5	−2.41	0.038	−0.22
Anger	1273.0	−4.71	<0.001	−0.43
Sadness	1717.5	−2.52	0.030	−0.23
Anxiety	1507.5	−3.62	<0.001	−0.33

**Table 8:** Yoga vs. SEL (H3): Δ-scores, Mann–Whitney U (Holm-adjusted p).

Variable (Δ)	U	Z	padj(Holm)	r
Overall stress	1882.0	0.73	0.46	0.04
Anger	1870.5	0.65	0.52	0.03
Sadness	1885.5	0.71	0.48	0.04
Anxiety	1895.0	0.77	0.44	0.05

**Notes.** Positive Z/r indicate relatively larger Δ in Yoga; negative values would indicate larger Δ in SEL. No contrast remained significant after Holm adjustment.

## Data Availability

The data presented in this study is available on OSF during peer review (private view-only); will be public upon acceptance at May, I. (4 June 2025). StressIntervention. Retrieved from https://osf.io/urfx7/?view_only=bc74a675a26f47ee81f0a6639d64eec3 accessed on 4 June 2025. Anonymized raw data, analysis scripts, and Supplementary Materials have been archived to ensure transparency and replicability.

## Supplementary Materials

The following supporting information can be downloaded at: https://www.mdpi.com/article/10.3390/bs15101374/s1. Supplement S1. Monitoring Instruments & Procedures. Instruments and procedures for process monitoring (attendance sheets, fidelity checklist, deviation log templates; scoring/decision rules; IRR metrics). No participant-level data are included; Supplement S2. Statistical Formulas and R Code. Effect-size formulas and fully annotated R code for Wilcoxon and Mann–Whitney tests, Holm adjustment within families, Hodges–Lehmann estimators with 95% BCa CIs (B = 2000), and aligned-ranks ANOVA on Δ-scores; includes sessionInfo.
