# Supplementary material for: Exploratory Pre–Post Study of School-Based Stress Interventions in Primary School Children
Source: Behav Sci (Basel). 2025 Oct 9;15(10):1374. doi: 10.3390/bs15101374 (PMC12561545; doi:10.3390/bs15101374)
Supplement: Supplementary file 1 [file behavsci-15-01374-s001.zip › Supplement S1.pdf]

Manuscript ID: behavsci-3855496
Title: Exploratory Pre–Post Study of School-Based Stress Interventions in Primary School Children?
Section: Supplement S1 — Monitoring Instruments & Procedures
Version/Date: v1.0 — 02 Oct 2025
Contact: isabelle.may@fau.de

Supplement S1. Monitoring Instruments & Procedures

Yoga • SEL • Therapeutic Climbing

Overview

This supplement documents the **instruments and procedures** used to monitor (a) session attendance, (b) implementation fidelity, and (c) protocol deviations. It does **not** contain participant-level data or numerical aggregates.

At a glance

- **Attendance:** per-session checklists completed by instructors.
- **Fidelity:** binary item checklist aligned with manuals; optional double-rating for agreement.
- **Deviations:** structured log with predefined categories, severity, corrective action, and resolution.

1) Attendance Monitoring

Instrument

- **Form:** session attendance sheet per class/date (one row per participant; one column per planned session).
- **Fields:** participant ID • date • session no. • present (1/0) • reason for absence (optional) • notes.

Recording Rules

- **Present = 1**, **Absent = 0**.
- If a **class-wide cancellation** occurs, the session is removed from denominators.
- Individual excused absences remain in denominators unless the session was **not offered** to that participant.

Denominators & Computation

- **Weekly denominators** reflect sessions actually scheduled for that arm.
- **Participant-week attendance %** = attended / planned × 100.
- **Arm-level summaries** aggregate over participants and weeks.

Data Governance

- Instructors complete sheets **immediately after** each session.
- Study lead reviews entries **weekly** for completeness and consistency.

2) Implementation Fidelity

Checklist Design

Binary ("Yes"/"No") items mapped to the scripted manuals. Typical items include:

- **General:** safety briefing; time-on-task as scheduled; materials prepared; closure/reflection conducted.
- **Yoga:** warm-up; breathing/centering; core sequence as scripted; cooldown/relaxation.
- **SEL:** opener/rapport; psychoeducation segment; guided practice; classroom transfer task.
- **Climbing:** equipment/safety check; instructor–student ratio; core climbing task; structured debrief.

Projects may use 5–10 items per session; wording mirrors the respective manuals.

Rating & Sampling

- Primary rater: **session instructor**.
- A subset (e.g., ~40%) is **double-rated** (second rater in situ or video-coded where permissible).

Scoring

- **Item-level adherence:** % "Yes" per item across sessions.
- **Mean adherence per arm:** mean across items and sessions (descriptive).
- **Trigger for deviation log:** any scripted element **not delivered** or substantial **timing/dose** change.

3) Protocol Deviations (Logging & Governance)

Definition

Any departure from protocol at class or session level, including:

Contact: isabelle.may@fau.de

- **Timing** (late start/shortened/overrun), **Content** (adaptation/omission), **Dose** (reduced/combined),
- **Safety** (equipment/ratio incident), **Logistics** (room change, space unavailable).

Log Fields

date • arm • class • week • session no. • deviation type • severity (minor/major) • reason • corrective action • resolved (yes/no) • notes.

Governance

- Instructors log deviations **same day**.
- Study lead reviews entries **weekly** and documents corrective actions.

4) Inter-Rater Agreement (for Double-Rated Sessions)

- **Metrics:** percent agreement and **Cohen's κ** on binary checklist items.
- **Scope:** computed across all double-rated sessions; item-wise estimates can be reported if needed.

5) Data Protection & Retention

- This supplement contains **no personally identifying information**.
- Participant-level IDs (if used in attendance logs) are stored separately and **not shared**.
- Monitoring documents are retained under institutional policy and applicable data protection law.
- **Availability:** On editorial request, **aggregate summaries** may be provided subject to data-protection constraints.

6) Abbreviations

SEL = Social and Emotional Learning; **κ** = Cohen's kappa.

Monitoring Instruments & Procedures — Fidelity Checklist Items

Manuscript ID: behavsci-3855496
Title: Exploratory Pre–Post Study of School-Based Stress Interventions in Primary School Children?
Section: Supplement S1 — Monitoring Instruments & Procedures
Version/Date: v1.0 — 02 Oct 2025
Contact: isabelle.may@fau.de

Scope. Binary session-level fidelity checklist (Yes/No/N/A) aligned with the scripted manuals for Yoga, SEL, and Therapeutic Climbing. Use short comments for any "No".

A. Core items (apply to all formats)

Rate **Yes (1)** / **No (0)** / **N/A**.

- **C1.** Session started within ±5 minutes of schedule.
- **C2.** Planned **dose** delivered (±10% of scripted duration).
- **C3. Safety briefing** and environment check completed.
- **C4. Materials/equipment** prepared and functional.
- **C5. Script adherence:** all key components delivered as manualized (no major omissions).
- **C6. Closure/debrief** conducted as planned (reflection / take-away).

Core fidelity score = mean of C1–C6 (excluding N/A).

B. Yoga add-on items (15-minute sessions)

- **Y1. Breathing/centering** delivered at session start.
- **Y2. Core sequence** delivered as scripted (structure and progression).
- **Y3. Adaptive options** safely offered (child-appropriate; "no pain" cueing).
- **Y4. Cooldown/relaxation** (e.g., guided relaxation) completed.

Yoga total fidelity = mean of C1–C6 and Y1–Y4.

C. SEL add-on items (45-minute sessions)

- **S1. Opener/rapport** activity implemented (check-in, norms).
- **S2. Psychoeducation** segment delivered as planned (target concept named).
- **S3. Guided practice** (e.g., role-play / skills rehearsal) completed.
- **S4. Transfer task** assigned or discussed (classroom/home application).

SEL total fidelity = mean of C1–C6 and S1–S4.

D. Therapeutic Climbing add-on items (60 min climbing + 30 min debrief)

- **CL1. Equipment & harness/belay check** documented.
- **CL2. Instructor–student ratio** meets protocol.
- **CL3. Progressive climbing task** delivered (difficulty/progression per manual).

- **CL4. Structured debrief** (30 min) using the prompt set completed.

Climbing total fidelity = mean of C1–C6 and CL1–CL4.

E. Scoring & decision rules

- **Yes = 1**, **No = 0**, **N/A** excluded from the denominator.
- **C2 (dose):** "Yes" if total session duration within **±10%** of the scripted dose (Yoga 15 min; SEL 45 min; Climbing 60 + 30 min).
- **C5 (script adherence):** "No" if a **core element** is missing or the sequence is substantially altered **without** pedagogical/safety justification.
- **C6/CL4 (debrief):** "Yes" if structured reflection prompts were used (≥5–10 min for Yoga/SEL; **30 min** for Climbing).

F. Inter-rater agreement (if double-rated sessions are used)

- Double-rate ~**40%** of sessions where feasible.
- Report **percent agreement** and **Cohen's κ** on the binary items (overall and, if desired, per item).

G. Deviation trigger & logging (links to the deviations log)

A **protocol deviation** is logged if any of the following occur:

- **Timing/Dose:** >10% deviation from planned duration.
- **Content:** a manualized **core element** omitted or replaced.
- **Safety/Logistics:** equipment/space issues, ratio violation, or unplanned room change.
  Log fields: date, arm, class, week, session no., deviation type, severity (minor/major), reason, corrective action, resolved (yes/no), notes.

H. Abbreviations

SEL = Social and Emotional Learning; **κ** = Cohen's kappa.
