# Supplementary material for: Exploratory Pre–Post Study of School-Based Stress Interventions in Primary School Children
Source: Behav Sci (Basel). 2025 Oct 9;15(10):1374. doi: 10.3390/bs15101374 (PMC12561545; doi:10.3390/bs15101374)
Supplement: Supplementary file 1 [file behavsci-15-01374-s001.zip › Supplement S2.pdf]

Manuscript ID: behavsci-3855496
Title: Exploratory Pre–Post Study of School-Based Stress Interventions in Primary School Children?
Section: Supplement S2 — Statistical Formulas and R Code
Version/Date: v1.0 — 02 Oct 2025
Software: R 4.4.0 (R Core Team, 2024); packages listed below.

Supplement S2. Statistical Formulas and R Code

1. Overview

Primary analyses use **rank-based nonparametric tests** with **Holm** control within pre-specified families. Effect sizes are reported with **95% bootstrap CIs**. Sensitivity checks include **Hodges–Lehmann (HL)** estimates and a **robust rank-based ANOVA on aligned ranks** for Δ-scores.

2. Data structure (expected tidy format)

A long/tidy data frame with at least:

- `id` (participant), `arm` ∈ {Yoga, SEL, Climbing},
- `outcome` (Overall stress, Anger, Sadness, Anxiety, or SWE),
- `time` ∈ {Pre, Post}, `value` (Likert 1–5).

For between-arm tests (H3), compute Δ-scores per participant: `delta = Post − Pre`.

3. Primary tests and multiple-testing control

- **H1 (Yoga)** and **H2 (SEL):** Wilcoxon **signed-rank** on paired Pre/Post.
- **H3 (Yoga vs. SEL):** Mann–Whitney **U** on Δ-scores (post–pre).
- **H4 (Climbing, exploratory):** Wilcoxon signed-rank on paired Pre/Post (SWE).

Holm (α = .05, two-sided) within families:
(a) Yoga within-group outcomes; (b) SEL within-group outcomes; (c) Yoga-vs-SEL Δ-comparisons. Climbing (H4) is exploratory (single outcome), no further multiplicity adjustment.

4. Effect sizes (definitions)

Manuscript ID: behavsci-3855496
Title: Exploratory Pre–Post Study of School-Based Stress Interventions in Primary School Children?
Section: Supplement S2 — Statistical Formulas and R Code
Version/Date: v1.0 — 02 Oct 2025
Software: R 4.4.0 (R Core Team, 2024); packages listed below.

Within-group (Wilcoxon signed-rank):

- *r* = *Z* / √*N*_{pairs}, where *Z* is the standardized test statistic.

Between-group (Mann–Whitney U on Δ):

- **Rank-biserial correlation**
  $r_{\mathrm{rb}} = \frac{2U}{n_1 n_2} - 1$(range −1…+1), using the U for the group coded as "higher".
  Equivalent probabilistic form: $r_{\mathrm{rb}} = P(X > Y) - P(X < Y)$(ties contribute 0.5 to *U*; with ties, $r_{\mathrm{rb}} \approx \delta$).
- **Cliff's delta (δ):**
  $\delta = \frac{\#(x>y) - \#(x<y)}{n_1 n_2}$. (Ties contribute 0.)

Optional (practice-oriented): Paired **Cohen's d_z**
$d_z = \frac{\bar{d}}{s_d}$, where *d* are paired differences.

5. HL estimators and CIs

- **Paired HL (within-arm):** median of subject-wise differences, $\mathrm{HL}_{\text{paired}} = \mathrm{median}(X_{post} - X_{pre})$.
- **Between-arm HL difference on Δ:** $\mathrm{median}(\Delta_{Yoga}) - \mathrm{median}(\Delta_{SEL})$.
- **CIs:** nonparametric bootstrap (BCa), *B* = 2,000 resamples (seed reported).

6. Sensitivity: aligned-ranks ANOVA on Δ

Use **aligned ranks** (ART) for Δ-scores with factor **arm** (Yoga vs. SEL) and outcome as grouping. Report omnibus test and, if applicable, Holm-adjusted contrasts.

Manuscript ID: behavsci-3855496
Title: Exploratory Pre–Post Study of School-Based Stress Interventions in Primary School Children?
Section: Supplement S2 — Statistical Formulas and R Code
Version/Date: v1.0 — 02 Oct 2025
Software: R 4.4.0 (R Core Team, 2024); packages listed below.

7. R code (ready to run)

7.1 Setup

```
# R 4.4.0
set.seed(2025)
# Install if needed:
# install.packages(c("dplyr","tidyr","rstatix","boot","effsize","ARTool"))
library(dplyr)
library(tidyr)
library(rstatix)   # wilcox_test, p_adjust, etc.
library(boot)      # bootstrap CIs
library(effsize)   # cliff.delta
library(ARTool)    # aligned rank transform ANOVA (art)
```

7.2 Helpers (effects, HL, bootstrap)

```
# Wilcoxon signed-rank effect size r = Z/sqrt(Npairs) via coin-like
standardization
wilcox_r <- function(x_pre, x_post) {
  wt <- wilcox.test(x_post, x_pre, paired = TRUE, exact = FALSE, correct = FALSE)
  # Approximate Z from p-value and sign from median change
  z <- qnorm(wt$p.value/2, lower.tail = FALSE)
  z <- sign(median(x_post - x_pre, na.rm = TRUE)) * z
  r <- as.numeric(z) / sqrt(sum(complete.cases(x_pre, x_post)))
  list(p = wt$p.value, V = wt$statistic, Z = z, r = r)
}

# Mann–Whitney rank-biserial r and Cliff's delta on deltas
mw_effects <- function(delta1, delta2) {
  wt <- wilcox.test(delta1, delta2, exact = FALSE, correct = FALSE)
  n1 <- sum(!is.na(delta1)); n2 <- sum(!is.na(delta2))
  # U from W (Mann-Whitney U equals W minus n1*(n1+1)/2 for group1 ranks)
  ranks_all <- rank(c(delta1, delta2), ties.method = "average")
  R1 <- sum(ranks_all[seq_len(n1)])
  U <- R1 - n1*(n1+1)/2
  r_rb <- 2*U/(n1*n2) - 1

  cd <- effsize::cliff.delta(delta1, delta2)$estimate  # Cliff's delta
  list(p = wt$p.value, W = wt$statistic, U = U, r_rb = as.numeric(r_rb), delta =
as.numeric(cd))
}

# Paired HL and BCa CI
hl_paired <- function(x_pre, x_post) median(x_post - x_pre, na.rm = TRUE)

hl_paired_boot <- function(x_pre, x_post, R = 2000, conf = 0.95) {
  dat <- cbind(x_pre, x_post)
  stat <- function(d, idx) {
    x <- d[idx, 1]; y <- d[idx, 2]
    median(y - x, na.rm = TRUE)
```

```
  }
  b <- boot::boot(data = dat, statistic = stat, R = R)
  ci <- tryCatch(boot::boot.ci(b, type = "bca", conf = conf)$bca[4:5], error =
function(e) c(NA, NA))
  list(HL = stat(dat, seq_len(nrow(dat))), CI = ci, boot = b)
}

# Between-arm HL difference on deltas (Yoga − SEL) with BCa CI
hl_between_boot <- function(delta1, delta2, R = 2000, conf = 0.95) {
  dat <- list(a = delta1, b = delta2)
  stat <- function(d, idx) {
    a <- d$a[idx[[1]]]; b <- d$b[idx[[2]]]
    median(a, na.rm=TRUE) - median(b, na.rm=TRUE)
  }
  # Paired resampling within arms
  stat_wrap <- function(dummy, i) {
    i1 <- sample.int(length(dat$a), replace = TRUE)
    i2 <- sample.int(length(dat$b), replace = TRUE)
    stat(list(a = dat$a, b = dat$b), list(i1, i2))
  }
  b <- boot::boot(data = matrix(0, nrow=1, ncol=1), statistic = function(d, i)
stat_wrap(d, i), R = R)
  ci <- tryCatch(boot::boot.ci(b, type = "bca", conf = conf)$bca[4:5], error =
function(e) c(NA, NA))
  list(HLdiff = median(dat$a, na.rm=TRUE) - median(dat$b, na.rm=TRUE), CI = ci,
boot = b)
}
```

7.3 Within-arm tests (H1, H2, H4) with Holm

```
# df_long: id, arm, outcome, time, value
within_results <- df_long %>%
  filter(arm %in% c("Yoga","SEL") & outcome %in% c("Overall
stress","Anger","Sadness","Anxiety")) %>%
  pivot_wider(names_from = time, values_from = value) %>%
  group_by(arm, outcome) %>%
  reframe({
    w <- wilcox_r(Pre, Post)
    hl <- hl_paired_boot(Pre, Post, R = 2000)
    tibble(
      test = "Wilcoxon signed-rank (paired)",
      n_pairs = sum(complete.cases(Pre, Post)),
      p_exact_or_approx = w$p,
      r = w$r,
      HL_delta = hl$HL,
      HL_CI_low = hl$CI[1],
      HL_CI_high = hl$CI[2]
    )
  }) %>%
  group_by(arm) %>%
  mutate(p_holm_within_family = p.adjust(p_exact_or_approx, method = "holm")) %>%
  ungroup()
```

Manuscript ID: behavsci-3855496
Title: Exploratory Pre–Post Study of School-Based Stress Interventions in Primary School Children?
Section: Supplement S2 — Statistical Formulas and R Code
Version/Date: v1.0 — 02 Oct 2025
Software: R 4.4.0 (R Core Team, 2024); packages listed below.

```
# Exploratory Climbing (H4: SWE)
climb_results <- df_long %>%
  filter(arm == "Climbing", outcome == "Self-efficacy (SWE)") %>%
  pivot_wider(names_from = time, values_from = value) %>%
  reframe({
    w <- wilcox_r(Pre, Post)
    hl <- hl_paired_boot(Pre, Post, R = 2000)
    tibble(
      test = "Wilcoxon signed-rank (paired)",
      n_pairs = sum(complete.cases(Pre, Post)),
      p_exact_or_approx = w$p,
      r = w$r,
      HL_delta = hl$HL,
      HL_CI_low = hl$CI[1],
      HL_CI_high = hl$CI[2]
    )
  })
```

7.4 Between-arm Δ (H3) with Holm + effect sizes

```
delta_df <- df_long %>%
  filter(arm %in% c("Yoga","SEL"), outcome %in% c("Overall
stress","Anger","Sadness","Anxiety")) %>%
  pivot_wider(names_from = time, values_from = value) %>%
  mutate(delta = Post - Pre)
between_results <- delta_df %>%
  group_by(outcome) %>%
  reframe({
    a <- delta[arm == "Yoga"]; b <- delta[arm == "SEL"]
    eff <- mw_effects(a, b)
    tibble(
      test = "Mann–Whitney U on Δ",
      n_yoga = sum(!is.na(a)), n_sel = sum(!is.na(b)),
      p_exact_or_approx = eff$p,
      U = eff$U,
      r_rank_biserial = eff$r_rb,
      cliffs_delta = eff$delta
    )
  }) %>%
  mutate(p_holm_within_family = p.adjust(p_exact_or_approx, method = "holm"))
```

7.5 HL difference on Δ with BCa Cis

```
hl_between <- delta_df %>%
  group_by(outcome) %>%
  reframe({
    a <- delta[arm == "Yoga"]; b <- delta[arm == "SEL"]
    hb <- hl_between_boot(a, b, R = 2000)
    tibble(
      HL_diff_YminusS = hb$HLdiff,
      HL_diff_CI_low  = hb$CI[1],
      HL_diff_CI_high = hb$CI[2]
    )
  })
```

Manuscript ID: behavsci-3855496
Title: Exploratory Pre–Post Study of School-Based Stress Interventions in Primary School Children?
Section: Supplement S2 — Statistical Formulas and R Code
Version/Date: v1.0 — 02 Oct 2025
Software: R 4.4.0 (R Core Team, 2024); packages listed below.

7.6 Sensitivity: aligned-ranks ANOVA on Δ (ART)

```
# Two-arm ART per outcome
art_results <- delta_df %>%
  select(id, arm, outcome, delta) %>%
  group_by(outcome) %>%
  reframe({
    fit <- ARTool::art(delta ~ arm)
    an <- anova(fit)
    tibble(
      outcome = unique(outcome),
      F_aligned = an$`F value`[1],
      df1 = an$Df[1],
      df2 = an$Df[2],
      p = an$`Pr(>F)`[1]
    )
  })
```

7.7 Putting it together (exportable tables)

```
# Table S4-like (HL within + between)
table_S4 <- within_results %>%
  select(arm, outcome, HL_delta, HL_CI_low, HL_CI_high) %>%
  mutate(Within = sprintf("%.2f [%.2f, %.2f]", HL_delta, HL_CI_low, HL_CI_high))
%>%
  select(arm, outcome, Within) %>%
  pivot_wider(names_from = arm, values_from = Within) %>%
  left_join(hl_between, by = "outcome") %>%
  mutate(Between_Yoga_minus_SEL = sprintf("%.2f [%.2f, %.2f]", HL_diff_YminusS,
HL_diff_CI_low, HL_diff_CI_high)) %>%
  select(Outcome = outcome, Yoga = Yoga, SEL = SEL, `Between (Yoga–SEL)` =
Between_Yoga_minus_SEL)

# Table S6-like (between-arm effect sizes)
table_S6 <- between_results %>%
  transmute(Outcome = outcome,
            `Rank-biserial r [95% CI]` = sprintf("%.2f [%.2f, %.2f]",
r_rank_biserial, NA, NA),
            `Cliff's δ` = sprintf("%.2f", cliffs_delta),
            `p (Holm)` = signif(p_holm_within_family, 3))

# Table S7-like (paired Cohen's dz, optional)
cohens_dz <- function(pre, post) {
  d <- post - pre
  sd <- sd(d, na.rm = TRUE); if (!is.finite(sd) || sd == 0) return(NA_real_)
  mean(d, na.rm = TRUE) / sd
}
table_S7 <- df_long %>%
  filter(arm %in% c("Yoga","SEL"), outcome %in% c("Overall
stress","Anger","Sadness","Anxiety")) %>%
  pivot_wider(names_from = time, values_from = value) %>%
  group_by(arm, outcome) %>%
  summarize(dz = cohens_dz(Pre, Post), .groups = "drop") %>%
```

```
  mutate(dz = sprintf("%.2f", dz)) %>%
  pivot_wider(names_from = arm, values_from = dz) %>%
  rename(Outcome = outcome)
# Export if needed:
# write.csv(table_S4, "S2_table_S4.csv", row.names = FALSE)
# write.csv(table_S6, "S2_table_S6.csv", row.names = FALSE)
# write.csv(table_S7, "S2_table_S7.csv", row.names = FALSE)
```
